# Hospital and Institutional News

**Published:** 1912-11-16

**Authors:** 


### HOSPITAL AND INSTITUTIONAL NEWS.

### A WOMEN'S AMBULANCE FOR THE FRONT.

On Monday night the first ambulance detachment sent out by the Women's Sick and Wounded Convoy Corps left Victoria for Sofia to proceed to the base hospital at Jambole, where the members will have charge of a ward placed at their disposal by the Bulgarian military authorities. We have frequently referred to this excellently trained and patriotically spirited corps of enthusiastic and energetic women, who have given four years to thorough training for field ambulance work. The Corps, which was started in 1908 and is recognised by the War Office and by the Red Cross Society, is perhaps one of the most efficient voluntary aid detachments in existence, and its offer to send a special unit to the front has been instantly accepted by the Bulgarian authorities. The unit that left on Monday night is composed of two women doctors, two trained hospital nurses who have had war experience, and eight members who are fully qualified in home nursing and first-aid and have had special training in hospital work. All of them have worked for several months in out-patient departments of some of the London hospitals, and have been initiated into the theory of war service at annual camps, at which they have had the benefit of special tests set by officers of the R.A.M.C. The Commandant of the Corps, Mrs. Stobart Greenhalgh, has preceded the detachment to Sofia, and is arranging for the work at that end; so that the work of expediting the departure of the detachment from London has fallen upon her able lieutenants, Miss Streatfield and Miss Gibbs. It may be mentioned that the detachment is in every sense of the word a volunteer unit: the members pay for their own outfit, and the expenses of equipment and stores have been defrayed by voluntary subscription. More money is needed, for the Corps intends to send out more nurses, and donations in aid of the special fund will be gratefully acknowledged by the Secretary at the Corps' Headquarters, Great Smith Street, Westminster.

### THE STRAIN ON THE RED CROSS SOCIETIES.

Only now that the numbers of wounded in the Balkan War are beginning to be known is the true extent of the need for Red Cross and Crescent activities capable of true apprehension. From all quarters are arriving appeals for further assistance. An urgent telegram arrived last week from the Crown Prince of Montenegro to the Medical Relief Committee of the British Red Cross Society, with the result that it was decided to dispatch to Antivari last Sunday three nursing orderlies and six women nurses, together with an equipment to the value of £200. Colonel C. Delmé-Radcliffe, the director of the Red Cross Services in Greece, has also received by telegram £500 for an immediate extension of the work. So far the Society has dispatched three units to Turkey, two each to Bulgaria and Greece, and one to Montenegro, and in the field at the present time are twenty-four surgeons and one hundred and twenty-six trained men, with full equipment for the work of attending to the wounded. To defray the expenses involved in this great effort the public had subscribed up to the end of last week some £20,500. Nor must it be supposed that the British Red Crescent Society has been less busy. Up to date it has dispatched to Turkey one hospital, under the direction of Colonel Surtees, the staff of which comprises two surgeons, three nurses, four orderlies, and six women nurses. A second hospital will be dispatched as soon as funds allow. A fresh appeal has, therefore, been issued by the Society, and subscriptions should be sent to Coutts and Co., the Society's bankers, 440 Strand, or to Mr. A. S. M. Anik, the Society's honorary treasurer, at 2 Fenchurch Avenue. From Egypt, it is reported, the advance division of the Egyptian Red Crescent Society left on Wednesday last week for Constantinople, and a further contingent sailed the following day.

### A GREEK HOSPITAL SENT TO THE FRONT.

Private munificence continues to play an important part in the equipment of field hospitals and Red Cross units for the Balkan War. One of the latest examples is the private base hospital which Mr. and Mrs. A. Embiricos have dispatched to attend to the Greek sick and wounded. The hospital is in charge of Dr. E. A. Cockayne, M.D., medical registrar to Middlesex Hospital, and Mr. C. H. S. Webber, F.R.C.S., surgical registrar to the Middlesex Hospital, who will act respectively as physician and surgeon in charge. The destination at present is Athens, but it is thought possible that the base hospital may have to be moved nearer the front. Mr. Embiricos, whose wife, by the way, is stated to be accompanying the party with a view to assisting in the management of the hospital, is himself a Greek shipowner. The nurses are English, like the medical men, who have been granted leave of absence from the Middlesex Hospital for three months.

### THE BRITISH RED CROSS SOCIETY IN SCOTLAND.

The Scottish Balkan unit organised by the Scottish Branch for service at the seat of war left Glasgow on Thursday night, and will leave London for Servia this, Saturday, night. This unit includes three medical officers—Major H. E. M. Douglas, V.C., D.S.O., who distinguished himself in the Boer War, being in command; and Dr. H. C. D. Rankin and Dr. C. Stewart Black, who are both graduates of Glasgow University. There are three dressers, five nursing orderlies, with five general duty orderlies, one sergeant, and one cook, or eighteen in all. Though great care has been taken in the selection of the men, both as regards health and fitness for duty, there has been no difficulty in getting a sufficient number. The dressers are all fifth-year medical students of Glasgow or Edinburgh. Some of the nursing orderlies are also medical students. The general duty men have each of them been trained in ambulance and first-aid work, and some possess a good knowledge of cooking. The cook holds the Aldershot certificate. Each man has a full personal equipment, with uniform, brassard, haversack (fitted), mess tin (Cavalry), water-bottle, clasp knife, sweater, three flannel shirts, three pairs of socks, two pairs of drawers, one blanket, one waterproof sheet, first field-dressing and kit bag. The unit takes with it at least one month's supply of provisions, with extra blankets and medical comforts. The medical and surgical equipment is the same as that published last week, see page 161. The cost of the unit is estimated to amount to £7,000, and a further sum will be necessary to provide warm clothing for the sick and wounded, the need for which will be intensified as the winter advances.

### AN AMBULANCE SERVICE FOR LONDON—THE DILATORY LONDON COUNTY COUNCIL.

In The Hospital of March 30, 1889, pages 403-4, and on page 12 of The Hospital for April 6, 1889, will be found full particulars of the origin of the street-ambulance service for London, with which the late Mr. H. L. Bischoffsheim's name will always be identified. As reported on the latter page, speaking in support of the organisation of this service, and announcing the receipt of the necessary money, Mr. Burdett said the question asked was whether the Council would do this work. His answer was that it might ultimately do so, but that while we were waiting for the County Council to find out what powers it possessed and how it could most usefully exert those powers, people were unnecessarily suffering and unnecessarily dying in London. Those words were uttered twenty-three years ago, and to-day we are able to report that the London County Council has at length taken action, which we hope means that serious steps may now be taken to provide an official ambulance service for London.

### WILL LONDON EVER SECURE AN EFFICIENT AMBULANCE SERVICE?

A Sub-Committee of the Council has had the question under consideration for a year or two and has gathered together a great amount of information on the subject. Particulars have been obtained as to the existing ambulances maintained by the Metropolitan Police, the Port of London Authority, the Metropolitan Asylums Board, the Borough Councils, the Boards of Guardians, and the various London hospitals. Having considered the matter very carefully, the General Purposes Committee came to the conclusion that there exists at the present time in London, material which, with efficient co-ordination and organisation, will furnish adequate ambulance service, and they conceive it to be the duty of the County Council to undertake such co-ordination. The Local Government Board, however, while approving of the principle of a united ambulance service for London, state that the public bodies which already have ambulances do not seem to be legally empowered to enter into agreements with the Council and they are advised that legislation would be necessary to secure the object in view. The County Council have accordingly decided to apply for Parliamentary powers to enable them to enter into agreements with other bodies for the use of their premises and ambulance appliances. The Metropolitan Police at present maintain 336 ambulances which are all available for use in street accidents, and the Council have decided to urge the Home Secretary to extend and improve the provision of ambulance appliances by the police. During the debate which took place upon the proposal, Mr. H. L. Jephson paid tribute to the work done by Sir Henry Burdett in his efforts to secure that such a service should be undertaken by the County Council.

### PROVINCIAL HOSPITAL LAUNDRIES.

We are indebted to Mr. J. A. Rudd, secretary to the Coventry and Warwickshire Hospital, and to Mr. Tom B. Harrison, secretary to the Chichester General Infirmary, for some interesting particulars of the working and cost of the laundries attached to their hospitals. In thanking these gentlemen for their contributions, we regret that pressure on space prevents us from publishing Mr. Harrison's paper this week. The new laundry at Coventry occupies a building which was formerly used as a cycle factory, and we have already commended—in The Hospital of December 25, 1909—the skill with which these buildings were adapted and altered for the purposes of a modern laundry. We hope to publish the plan of this laundry next week, as its simplicity and ease of working may make it of use to some of the smaller hospitals. The Coventry and Warwickshire Hospital contains eight-five beds, and the laundry, including buildings and equipment, cost £5,340.

### THE SANATORIUM CONTROVERSY.

Sanatorium benefit still continues to excite criticism, debate, and official disclaimers. Nor is it only to its alleged defects of administration that attention is being angrily directed. The latest critics are the tuberculin enthusiasts, and among them Dr. Mary Hamilton Williams, who declares that Mr. Lloyd George's suggestion of payment by a capitation fee of 6d. per annum in place of payment for work done will destroy "all that was best in the original scheme." The alteration, she continues, will militate against the early recognition of the disease, and by the small fees it offers will tend to discourage practitioners from diagnosing tuberculosis, because they will, she says, realise that such diagnosis would involve very laborious treatment for very small remuneration. Practitioners will thus, she believes, be inclined to recommend sanatorium treatment in almost every case. She urges further that sanatorium treatment will be far more costly than domiciliary tuberculin treatment, while in conclusion she makes the inevitable assertion of the tuberculin enthusiast that statistics on actuarial lines show that the permanent results of sanatorium treatment are not at all commensurate with the expense involved. With the earlier statements we are not for the moment concerned, but we think it well to remind Dr. Williams and others of her way of thinking that with all its defects sanatorium treatment remains the only one which has stood the test of fifteen years. Tuberculin, it is not heresy to say, has not yet so well proved its claims as to be free from the charge that it may join the already too long list of consumption panaceas that have had to be abandoned. In the sphere of consumption therapeutics fifteen years is an exceptional record.

### THE INSURANCE ACT.

Exactly what effect the recent defeat of the Government over the finance of the Home Rule Bill will have upon the political situation remains to be seen; but, obviously, if there were to be a change of Government before January 15, the whole question of medical benefit would have to be thrashed out afresh with whomsoever should occupy Mr. George's shoes. For the moment, however, it seems safe to conclude that no such change is imminent, and to proceed upon that assumption. The signs are demonstrating that the new offer of the Chancellor to the medical profession over medical benefit will be refused by the British Medical Association. This we regret; for, although we do not regard the new offer as wholly satisfactory, we do think it could be made the basis for an arrangement which would prove equitable to the profession. This view was expressed in these columns at the time the new offer was made, and we have as yet seen no sufficient reason for changing it (The Hospital, November 2, 1912). The suggested powers of inspection which Mr. George proposed still seem to be very repugnant to many members of the profession; for the life of us we cannot see why, provided that such inspection is carried out by medical men and its results reported to a board or committee of medical men. It would, of course, be harassing and unfair to have the inspection controlled by friendly society officials or other laymen; but the profession would do much better to bargain for a system of control which will give them justice and fair dealing than to reject the principle with contumely.

### THE TREND OF OPINION AMONG DOCTORS.

So far as can be judged by reading the letters which the doctors themselves write to the *Lancet*, the *British Medical Journal*, the *Times*, and similar influential organs, there is some serious risk of a partial split in their ranks. This, no doubt, was anticipated by that master of Machiavellian tactics, Mr. Lloyd George. To begin with, the decisions of the branches of the British Medical Association have not been unanimous, as they were for all practical purposes over the original scheme. It is true that the bulk of the local meetings have voted a refusal of the suggested terms; but not all have done so, and a certain number of dissentients have showed up at the meetings where refusals have been carried. In a word, the profession is no longer solidly united, though we believe that there is a very substantial majority indeed against the new terms. Then, again, it is apparent that the mileage question is creating a certain amount of ill-feeling. Country doctors are not hesitating to say that they must have the lion's share of this, and even to hint that by means of it they can make serious inroads upon the money available for remunerating the town doctors. This very point was in our mind when we advised the doctors, a fortnight ago, to agitate for the provision of mileage out of an entirely separate fund, so that the promised six shillings and sixpence shall be guaranteed, clear of all charges upon it. As we predicted, the new provisions about record keeping and modern methods of diagnosis turn out to be very harmless; this much has been extracted out of Mr. George and Mr. Masterman by questions in Parliament. We shall be very sorry if the British Medical Association breaks off all negotiations again; but it looks as if that is what will happen.

### ENLARGEMENT OF THE MILLER HOSPITAL, GREENWICH.

The new wing recently in course of construction at this hospital is to be opened by Princess Louise, Duchess of Argyll, on Friday, November 15. It provides, in addition to the accommodation of the older buildings, a women's medical ward, a men's and a women's surgical wards, of sixteen beds each, and a small isolation room of one bed. An operating theatre also forms part of the new extension. All these additions to the hospital premises have been constructed in accordance with modern requirements, and the result as far as can be foreseen will be a great improvement in the way of accommodation upon anything which the Miller General Hospital has been able to offer hitherto. Three additional houses facing Greenwich Road have been taken to accommodate the extra staff needed for these new wards. Unfortunately it proved to be beyond the available resources to build a home actually designed for its purpose. All meals are served in the main building, not in the home, the dining room for the nursing staff being at the top of the hospital, adjacent to the kitchen.

### THE NEW WINGS AT WORKSOP HOSPITAL.

The opening of the two new wings at the Victoria Hospital, Worksop, took place last week by the Duchess of Portland and Sir John Robinson. It will be remembered that the first wing was undertaken as a Coronation Memorial, and the second has been the generous gift of Sir John Robinson, in memory of his late wife. Speaking later in the day the Duke of Portland drew attention to the need there had been of late for an extended accommodation, which the new buildings have virtually doubled. Mr. A. H. Richardson is the architect. The problem before the local hospital supporters is obviously now that of a greatly increased cost of maintenance. The new addition to the number of beds, both for adults and children, was badly needed, but it has necessitated a larger staff, and there is still a debt of £552 on the building. The donors of the Coronation and Children's Wings have, however, set such a generous example that it is felt that the further money needed will be forthcoming.

### CORNWALL'S NEW COTTAGE HOSPITAL.

THE new cottage hospital at St. Austell, Cornwall, which, as was noted at the time, has lost, up till the present at any rate, a legacy of £500 through its solicitor inadvertently filing its claim one month after the legal period had expired, was opened last week by Lady Layland-Barratt. The architect is Mr. R. Wheatley, and the institution is designed to serve the district largely occupied by men engaged in the china-clay industry, who have felt for a long time the need of a hospital, and who really have the credit of having started the new hospital. The chairman of the committee is Mr. H. S. Hancock. The cost of the building has worked out at almost £3,000, towards which £2,300 have been subscribed, while there are still hopes of the above-mentioned legacy, which will leave approximately £250 to be raised. Great pains were taken to give the hospital a good send-off; and, though the weather was unpropitious, a procession, a public tea followed by speeches, a concert, and a dance were all included in the proceedings of the day.

### A HOSPITAL FOUNDRESS IN VENICE.

THE death of Lady Layard at an advanced age in Venice removes one who was not only a very great lady of the Victorian era, but also the foundress of a hospital which has done a great deal of good for many years. Of all her many good works, says a correspondent of the *Times*, the one that lay nearest her heart was the Hospital for British Sailors, which she founded and supported by her exertions. In this charming home with its pretty garden on the banks of the Lagoon at Venice sailors who had sailed under the English flag were carefully tended by English nurses and skilled physicians, while others who could afford to pay were received in rooms set apart for this purpose; no English man or woman who was sick or destitute was ever turned away. There is a considerable British colony in Venice, of which Lady Layard was the acknowledged *doyenne* and social leader. It is to be hoped that among the leisured and wealthy of our countrymen who make their homes on the Grand Canal and its affluents some one or more will come forward to carry on the work in which Lady Layard took such an interest, and that the Hospital for British Sailors will continue to flourish as it did under the loving care of its foundress. It is also worthy of passing note that the death of Lady Layard brings to the National Gallery the reversion of Sir Henry Layard's magnificent collection of pictures, whose care (Lord Bessborough states) was always considered by his widow as one of her most sacred trusts.

### THE LATEST SEPARATED INFIRMARY.

IT would seem practically certain that the Brighton Board of Guardians are going to follow the line of progress adopted on a considerably wider scale by all the really efficient Boards and to separate the infirmary from the workhouse. Separation, at least, is what the report of the Special (Workhouse) Committee on the reorganisation of the medical administration of the workhouse really means. They suggest an infirmary self-contained from the medical point of view, to be placed entirely under the superintendence of the medical officer. The staff would consist of a senior officer, known as the medical superintendent of the infirmary, who would be its administrative head, and therefore a resident and whole-time official. He would live in a residence approved by the Guardians in close proximity to the workhouse. The salary suggested is £450, inclusive of all fees, together with £100 a year while non-resident. Among the duties of the medical superintendent, on which special stress is laid, are that he shall see that no person is retained in the wards because he or she is useful; that he shall examine each infant daily in the nursery and every child in the Warren Farm Schools at least once every three months. The committee are of opinion that, though extensive alterations and rearrangements of wards would be necessary, no new buildings would be immediately required, and that the separated infirmary possessing 300 beds would meet their requirements. The post of visiting-surgeon under this scheme would not be necessary. The chairman of the committee which has issued this report is Mr. C. J. Galliers, and Dr. Charles Fraser, Councillor A. L. Tindall, Mr. A. E. Mellor, and Mr. Henry Thomas were its other signatories. While various details, including the medical superintendent's salary, are open to criticism, the fact that a separated infirmary is the upshot of the report makes us inclined to lay little stress on these points. For separation is the only true basis of advance.

### THE PUTNEY HOSPITAL FOR INCURABLES.

THE usual notices have been issued convening a meeting of the subscribers to this invaluable charity at the Cannon Street Hotel on November 29. As usual twenty-five vacancies for pensions and for places in the hospital are available; and the accustomed system of vote collecting and counting will decide which of the 215 candidates will receive the benefits desired. We do not propose at present to say anything about this system, though it is far from being an ideal method of administering the charity. Like hospital letters it has inherent drawbacks, which the board of management doubtless fully realise; but, unlike the case at the general hospitals, the abolition of the system without interfering with the income of the charity is probably very difficult if not impossible. The list of candidates and of the causes of their impaired health presents features common to those of previous lists; but there are several very striking features about this catalogue of misfortune which are worthy of a brief examination. The pitiful wreckage of life here exhibited implies a wastage of human health and energy which is perfectly appalling.

### THE INCURABLE DISEASES OF THE CANDIDATES.

FIRST on the list of causes of disability comes "rheumatoid arthritis," which accounts for sixty-one cases, not counting seven or eight labelled "chronic rheumatism." Almost all the sufferers are women. This crippling complaint bulks more largely on the list than others not only because it is so common, but also because of itself it is not deadly; it disables its victims for years without greatly shortening their lives. Heart disease, on the other hand, accounts for surprisingly few cases: probably the majority of such patients only fail to earn their living a few months before their final breakdown, and the process of gaining admission to the hospital proves too long for them. Amongst the men locomotor ataxia is one of the common diagnoses; we should be the last to suggest that this disease should debar its victims from help and sympathy, but it does seem hard that tabetics should deprive of assistance others whose illnesses are the result of misfortune solely. Many of the diagnoses are extremely vague: "paralysis" is an instance, of which the list contains forty-six examples. This term might cover anything from alcoholic neuritis to a fractured spine. "Internal trouble" is another diagnosis which recurs three or four times. Of two patients with malignant disease, one is trying for the third time and the other for the ninth. The latter implies a duration of four and a-half years, and may therefore indicate an error in diagnosis. "Gastroenterostomy" is said to be the disease of one patient, a hospital nurse: how this can be is difficult to understand, but doubtless the poor lady's case is a sad one. Hæmophilia, Graves' disease, and many other serious maladies figure in this catalogue of human woe. If more help were forthcoming, the board of management could do more than ever to relieve the sad cases brought to their notice.

### PATIENTS AS APPEAL WRITERS.

On more than one occasion we have been able to praise the originality and care taken by the Royal Hospital for Incurables on Putney Heath in the matter and presentment of the appeal which the institution is accustomed to issue about this time every year. In 1910, if we remember, a capital speech by Charles Dickens on the claims of its patients was republished in pamphlet form, and we then noted with pleasure that the authorities had made a search for ideas in their own archives rather than elsewhere. That policy, which is far the best, for every institution has its individual possessions if only the will is there to find and exploit them for appeal purposes, has been carried yet further on the present occasion in the form of an attractive volume entitled "Thoughts of an Incurable," which proves on examination to be a calendar with "a thought" attached for every day in the year. Archdeacon Sinclair has provided an introduction. Such calendars from single authors are very popular nowadays: indeed, a first-rate one, like the Oscar Wilde Calendar for instance, practically took the place of the Christmas card among the immense numbers of his admirers when it first came out. The authorship of these "Thoughts" is anonymous, but many people will feel that we could give them no higher praise than to say that they might have been written by Mr. Arthur Christopher Benson. On March 5 is written: "Groans and tears are not wanted. Hospital visitors prefer to be cheered up." With that hint as to its contents we commend the "Thoughts of an Incurable" to the public, and congratulate the hospital on its good fortune in being able to minister to a patient whose work on its behalf is likely to carry the name of the Royal Hospital for Incurables on Putney Heath still further afield.

### A NEW EXCUSE FOR WITHDRAWING SUBSCRIPTIONS.

There is a certain class of subscribers to hospitals which is always glad of an excuse for "not sending a subscription this year," but it must be admitted that the reasons adduced do not generally have either the charm or force of novelty. Here, however, so far as we are aware, is an original variation of the Insurance Act argument: "I intend withholding my annual subscription this year," says a writer in a Midland daily newspaper, "until I see the attitude which the medical profession intend to adopt towards the local hospitals, from which they derive a large percentage of the knowledge subsequently disposed of at a big profit in their private practice. If the local medical men who attend the hospitals decline their services to persons compulsorily insured, then no subscription shall go to such hospitals." It is hardly worth while to point out that if this person's further suggestion that "a similar attitude be adopted by all subscribers" really became general, State or rate hospitals would follow, to the enrichment of the medical staffs, which would be able to exact high fees for their work. And, we fear, the subscriber's voluntary annual subscription would be replaced by a compulsory hospital rate on a probably much more generous scale. The proposal itself is so foolish that no one will be surprised to learn that it emanated from an anonymous correspondent.

### £300 A YEAR FOR A MEDICAL SUPERINTENDENT.

The London County Council has been considering a proposal that the officer in charge of the Farmfield Reformatory should be a medical man. The institution is certified for the reception of 113 female inebriates and is at present under the administrative charge of the Clerk of the County Council. A lady superintendent is the principal officer at the institution, while the medical side of the work is performed by a visiting medical practitioner. For some time past the Public Control Committee have felt that the responsibilities are heavier than a woman can be expected to cope with successfully, and it is therefore proposed to place a fully qualified man in charge of the reformatory. When the matter was discussed by the Council an amendment that the officer in charge should be a fully qualified man or woman was defeated. The whole matter was, however, referred back on the important question of salary. The Committee proposed £300 a year with fire, light, and residence. This luckily met with strong opposition, on the obvious ground that the best candidates would not apply. The question of salary is accordingly to be reconsidered.

### OPHTHALMIA NEONATORUM IN SCOTLAND.

A careful report has been issued, communicated to the Local Government Board for Scotland, by Dr. T. F. Dewar, one of the Board's medical inspectors, upon the incidence of ophthalmia amongst the new-born in Scotland and the measures advisable for its reduction. A large number of very interesting statistics are quoted, and the investigation discloses facts which can be briefly summarised. Ophthalmia neonatorum is less prevalent in Scotland now than it was twenty-five or even ten years ago; and it is less often followed by actual loss of sight. Nevertheless, the disease is not uncommon, and does account for many cases of blindness annually. Approximately, Dr. Dewar estimates, about 500 cases a year occur in Scotland, at least ten of which end in blindness, while thirty to forty more suffer impairment of sight. The majority of the experts whom Dr. Dewar consulted were in favour of making the disease compulsorily notifiable; but, as this view was not unanimous, he suggests that the facts concerning the prevalence, preventability, and dire results of it should be brought to the notice of local authorities and further action left to their initiative. Finally, a very general desire prevails amongst obstetric and ophthalmic practitioners in Scotland to have in that country a Midwives Act on lines resembling generally the English Act.

### LADY BROWNLOW'S WILL.

The passing generation at any rate shows little sign of faltering in its regard for the voluntary hospitals, and to the already famous list of rich hospital benefactors must be added the name of Lady Brownlow, the terms of whose will have now been declared. Dame Brownlow, to use the strict nomenclature of the law, left estate of £300,000, and after various bequests bequeathed the residue of her property to her husband for life, with remainder almost exclusively to hospitals. Thus King Edward's Hospital Fund for London will receive £20,000, and the Royal Berkshire Hospital and St. Mary's Hospital, Paddington, £2,000 each, while St. George's Hospital, London, the Royal Hospital for Incurables, and St. Andrew's Convalescent Home at Folkestone are to benefit by £1,000 in each case. In addition, the London Fever Hospital, the Plaistow Hospital, whose staff have been working so hard for their One Million Pennies Fund, and the East London Hospital for Children will each receive £500. Her total hospital bequests, therefore, amount to little short of £30,000, which should encourage not merely the already mentioned institutions but hospitals generally to believe that, however legislation may affect the destination of gifts during life, hospitals are still likely to retain the affectionate remembrance of testators.

### DIFFICULTIES FEARED AT ACTON.

The Cottage Hospital and Nursing Institution in Gunnersbury Lane, Acton, was founded to commemorate the Diamond Jubilee of Queen Victoria, and was rendered possible by the generosity of the late Mr. Passmore Edwards, who defrayed the cost of the building, and of Lord Rothschild and Mr. Leopold de Rothschild, who gave the site and have ever since aided its upkeep by generous donations. Unfortunately, however, the residents of the district have never fully realised the immense value of such a beneficent institution in their midst, with the result that its position financially has each year been a matter of considerable anxiety to the governing body. Some idea of the difficulty of just being able to "make ends meet" may be gathered from the honorary treasurer's report at the last meeting, when there was a balance at the bank of £90, but in reality only £10 in hand, as just £80 was required to be paid out at once. On the top of this came a speech by Mr. H. J. Thornton pointing to the "very serious time ahead' in consequence of the Insurance Act.

### HOSPITALS OR SCHOOL CLINICS.

The London Education Committee have now made their arrangements for the medical treatment of school children during the coming year. The Board of Education have requested that the arrangements at the other hospitals shall be brought into line with those at the London Hospital, where a system of "following-up" is in operation and where an inspection centre has been provided near the hospital. It is proposed to inform the Board that the arrangements for "following-up" at St. Mary's Hospital are similar to those at the London Hospital, while Charing Cross Hospital, the Belgrave Hospital for Children, and the Miller General Hospital will submit proposals on the desired lines. The establishment of one or two school clinics under the direct management of the County Council was urged by Mr. R. A. Bray, who said that there was very little difference between such clinics and the arrangements in force at the London Hospital. The proposal, however, was defeated.

### THIS WEEK'S DRUG MARKET.

With the exception of a demand for opium and its alkaloids and a few other drugs business has again been quiet. The value of opium is well maintained, but surprise is felt that there has been no marked and rapid advance in the price of this drug up to the present; the general opinion is that the value is sure to rise not only on account of the difficulties of transport but also because of the dislocation of labour in the producing districts. Quicksilver has been reduced, and in consequence makers of calomel, corrosive sublimate, and other salts of mercury have announced a reduction in their quotations. As was not unexpected there has been an advance in the price of cocaine. Balsam tolu is tending lower in value. Eucalyptus oil still tends dearer and is in good demand. Menthol still tends upwards in price. It is not altogether improbable that the price of bromides may be advanced before the end of the year. Saffron is dearer and the price of seidlitz powder has been advanced. The English makers of chemically pure glycerine decided on November 12 to raise their quotations.

